# CLICK-FLISA Based on Metal–Organic Frameworks for Simultaneous Detection of Fumonisin B1 (FB1) and Zearalenone (ZEN) in Maize

**DOI:** 10.3390/bios14070355

**Published:** 2024-07-21

**Authors:** Jingyang Zhang, Banglei Zhu, Xiaoyu Zhang, Yuan Peng, Shuang Li, Dianpeng Han, Shuyue Ren, Kang Qin, Yu Wang, Huanying Zhou, Zhixian Gao

**Affiliations:** Military Medical Sciences Academy, Academy of Military Sciences, Tianjin 300050, China; 15760527290@163.com (J.Z.); z1095317236@163.com (X.Z.); dalidao@139.com (Y.P.); liza3320@163.com (S.L.); 15210520025@126.com (D.H.); renshuyue2018@163.com (S.R.); qinkang2020@foxmail.com (K.Q.)

**Keywords:** food safety, mycotoxins, metal–organic framework, immunoassay

## Abstract

Mycotoxins are secondary products produced primarily by fungi and are pathogens of animals and cereals, not only affecting agriculture and the food industry but also causing great economic losses. The development of rapid and sensitive methods for the detection of mycotoxins in food is of great significance for livelihood issues. This study employed an amino-functionalized zirconium luminescent metal–organic framework (LOF) (i.e., UiO-66-NH_2)_. Click chemistry was utilized to assemble UiO-66-NH_2_ in a controlled manner, generating LOF assemblies to serve as probes for fluorescence-linked immunoassays. The proposed fluoroimmunoassay method for Zearalenone (ZEN) and Fumonisin B1 (FB1) detection based on the UiO-66-NH_2_ assembled probe (CLICK-FLISA) afforded a linear response range of 1–20 μmol/L for ZEN, 20 μmol/L for FB1, and a very low detection limit (0.048–0.065 μmol/L for ZEN; 0.048–0.065 μmol/L for FB1). These satisfying results demonstrate promising applications for on-site quick testing in practical sample analysis. Moreover, the amino functionalization may also serve as a modification strategy to design luminescent sensors for other food contaminants.

## 1. Introduction

Zearalenone (ZEN) and Fumonisin B1 (FB1) are mycotoxins that have been identified as the contaminants in grains, cacao beans, and meat products [1], and these contaminants may induce acute and chronic effects. Acute effects are characterized by a rapid onset of poisoning, and even death, while chronic effects include chronic pathological reactions of visceral functions and physiological process damage, such as carcinogenesis [2], teratogenesis [3], and mutagenesis [4]. Fungal contamination of food is a major source of mycotoxin poisoning, and such contaminants cannot be effectively eliminated through conventional cooking heat treatments [5]. Traditional detection methods, such as High-Performance Liquid Chromatography (HPLC) [6], are widely employed for mycotoxin detection due to their accuracy and sensitivity. However, the complexity and high cost of the equipment limit their applicability for rapid detection. An enzyme-linked immunosorbent assay (ELISA) [7] can detect mycotoxins simply and specifically [8]. Nevertheless, the deficiencies of ELISAs are also obvious [9], such as their lower sensitivity and susceptibility to external conditions. A fluorescence-linked immunosorbent assay (FLISA) retains the advantages of ELISAs while enhancing sensitivity and stability, and it has been widely studied in recent years [10].

Given that the quality of fluorescent labeling significantly influences FLISA detection performance, considerable effort has been dedicated in recent years to developing superior fluorophores [11,12,13].

Fluorescent nanomaterials, which possess fluorescent properties at the nanoscale, have significantly advanced the development of FLISAs, endowing them with superior characteristics. Many studies have demonstrated that the advent of novel fluorescent nanomaterials, such as semiconductor quantum dots [14], carbon dots [15], carbon nanoparticles [16], graphene-based nanomaterials [17], and metal–organic frameworks (MOFs) [18], has markedly enhanced the operational ease and sensitivity of FLISAs [19]. Among the reported fluorescent nanomaterials, MOFs have attracted noteworthy attention because of their structural tunability, surface modifiability, and excellent biocompatibility. A luminescent metal–organic framework not only has the advantages of MOF but is also easily able to induce luminescence, which is a potential fluorescent chemical sensor [20,21]. The luminescence of MOFs can be emitted by either organic linkers or metal ions. The linkers of central luminescence also include three subtypes: linker emission, ligand–metal charge transfer (LMTC), and metal–ligand charge transfer (MLCT) [22].

As a novel porous composite material, LMOFs can be effectively tailored by customizing their building blocks with specific recognition moieties, allowing them to selectively recognize and interact with target molecules for precise detection purposes [23]. Today, LMOFs have emerged as an ideal solution for mitigating food safety risks, finding extensive applications in detecting antibiotics [24], food additives [25], harmful ions [26], pesticide [27], and mycotoxins [28,29].

In recent years, numerous LMOF sensors for detecting mycotoxins in food have been designed to be sensitive to pH changes, as mycotoxins in food often lead to significant pH variations [28,30]. However, this method has two major drawbacks. It cannot differentiate between multiple targets, as various food hazards often coexist. Furthermore, pH-sensitive LMOF sensors typically have high detection limits, limiting their effectiveness in detecting real samples. For instance, Guo et al. reported a detection limit of 15 μM for 3-nitropropionic acid in sugarcane juice [28]. By contrast, this study innovatively integrates an LOMF with FLISA technology to revolutionize the conventional mycotoxin detection methods of LMOF sensors, significantly lowering the detection limit.

Hence, UiO-66-NH_2_ was selected for this study due to its well-documented superior properties, including high stability, good biocompatibility, and tunable structure. UiO-66-NH_2_ has demonstrated efficient fluorescence performance in various applications, particularly in sensing and bio-detection. While other MOFs may exhibit higher quantum yields, UiO-66-NH_2_ offers unique advantages that make it especially suitable for the specific applications in this research.

Although MOFs have shown remarkable potential in FLISAs, particularly in enhancing sensitivity and selectivity, their application prospects are extensive and varied; achieving rapid and effective conjugation of MOFs with antibodies remains a challenge. The development of click chemistry promotes the application of MOFs in chemical biology and could address the challenge effectively [31]. Among the various click chemistry reactions, the Cu(I)-catalyzed cycloaddition of azides and terminal alkynes (CUAAC) [32] is highly favored by researchers due to its simplicity and high reactivity. However, to circumvent the potential biological toxicity associated with Cu(I) salts, the inverse electron-demand Diels–Alder (IEDDA) [33] cycloaddition between 1,2,4,5-tetrazines (s-tetrazines, Tz) and trans-cyclooctene (TCO) was developed for labeling complex biological systems [34]. Therefore, a copper-free TCO-Tz reaction was employed to assemble the components for fluorescence immunoassay.

In this study, a novel fluorescence-linked immunosorbent assay (FLISA) was developed for the rapid and simultaneous detection of ZEN and FB1 in maize. The luminescent MOF was assembled using click chemistry to amplify the fluorescence signal and was subsequently conjugated to the secondary antibody. Thus, an immunoassay capable of directly generating fluorescent signals was developed. Compared to ELISAs and FLISAs, which use fluorescein isothiocyanate (FITC) as a tracer, the immunoassay developed in this study demonstrated superior performance when applied to the same group of corn samples.

## 2. Materials and Methods

All holoantigen (ZEN-BSA, FB1-BSA) murine monoclonal antibodies against ZEN and FB1 were purchased from Beijing Itest Technology Co., Ltd. (Beijing, China). ZEN standard solution, FB1 standard solution, deoxynivalenol (DON) standard solution, ochratoxin A (OTA) standard solution, Aflatoxin B1 (AFB1) standard solution, and Aflatoxin M1 (AFM1) standard solution were purchased from J&K Scientific Co., Ltd. (Beijing, China). Goat anti-Mouse IgG H&L (FITC) were purchased from Beijing Bioss Biotechnology Co., Ltd. (Beijing, China). Goat anti-Mouse IgG were purchased from Beijing Solarbio Technology Co., Ltd. (Beijing, China). MethylTetrazine-PEG4-NHS Ester and TCO-PEG4-NHS Ester were purchased from Xi’an Ruixi Biological Technology Co., Ltd. (Xi’an, China). N,N-dimethylformamide (DMF), 2-amino terephthalic acid, glacial acetic acid, and zirconium chloride (ZrCl_4_) were purchased from Aladdin (Shanghai, China). Phosphate-buffered saline (PBS, pH 7.4, 0.01 M) and sodium hydroxide (NaOH), methanol, and all other reagents were obtained from Macklin Biochemical Technology Co., Ltd. (Shanghai, China).

### 2.1. Preparation of MOFs

A total of 52.45 mg of ZrCl_4_, 40.75 mg of terephthalic acid, and 6 mL of acetic acid were dissolved in 50 mL of DMF. After 5 min of ultrasonication, the mixed solution was transferred into a reactor. The solution was then placed in an electric blast-drying oven at a constant temperature of 120 °C for 24 h. After the reaction, the reactor was gradually cooled to room temperature. The resulting solid–liquid mixture was centrifuged three times with N,N-dimethylformamide and methanol. Finally, white powder UiO-66 was obtained by drying overnight in an electric vacuum-drying oven at 90 °C. By replacing terephthalic acid with 2-amino terephthalic acid while keeping other experimental conditions the same, pale-yellow powder NH_2_-UiO-66 was obtained.

### 2.2. Preparation of CLICK-MOF Assemblies

**Figure 1:**
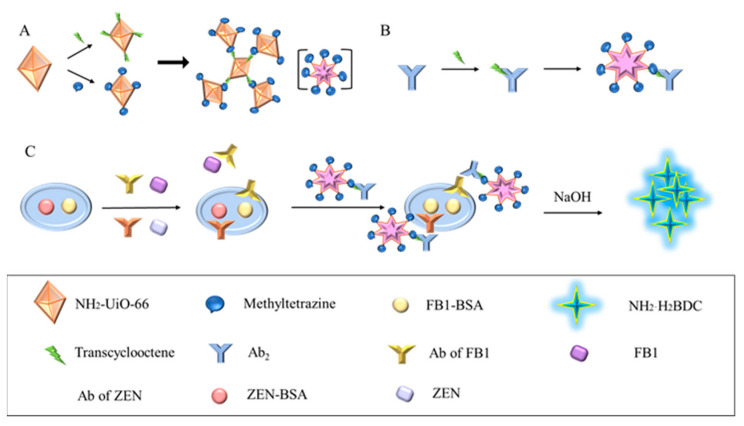
Schematic diagram of CLICK-FLISA detection for the target. (**A**) Synthesis of CLICK-MOF assemblies. (**B**) The coupling principle of TCO-Ab_2_ and CLICK-MOF assemblies. (**C**) CLICK-FLISA were formed, and the signal was read by a multifunctional microplate reader.

**Figure 2:**
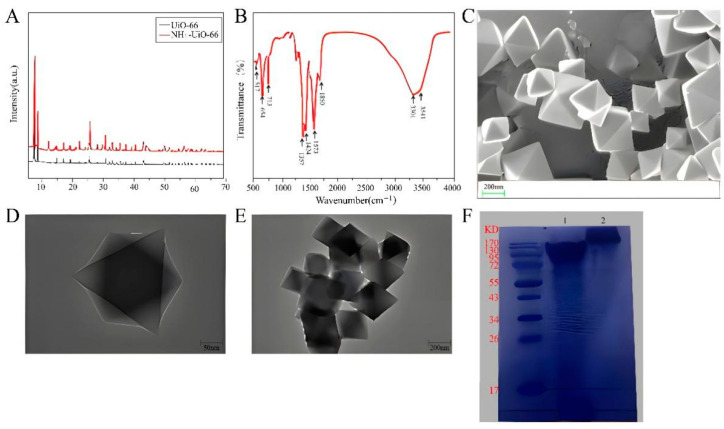
Feasibility of CLICK-FLISA strategy. (**A**) XRD spectra of synthetic UiO-66 and NH_2_-UiO-66. (**B**) FT-IR spectrum of synthetic NH_2_-UiO-66. (**C**) SEM image of synthetic NH_2_-UiO-66. (**D**) TEM image of synthetic NH_2_-UiO-66. (**E**) TEM image of CLICK-MOF assembly. (**F**) Protein gel electrophoresis of the TCO-Ab_2_.

Both TCO-PEG4-NHS Ester and methylTetrazine-PEG4-NHS Ester were dissolved to a concentration of 10 mM in DMF, and NH_2_-UiO-66 was diluted with PBS (pH 7.4, 10 mM). Next, the click ligand and aminoated metal–organic framework (NH_2_-UiO-66) were mixed in a molar ratio of 20:1, and the reaction was conducted at 37 °C for 1 h. The chemical equations and reaction principle for TCO-PEG4-NHS Ester and methyltetrazine-PEG4-NHS Ester are as shown in Figure S1. After the reaction, 50 mM Tris-HCl was added (pH = 8.0) to stop the reaction for 10 min. The mixed solution was centrifuged at 10,000 rpm for 5 min at 4 °C and washed 3 times with PBS (pH 7.4, 10 mM) to remove excess TCO and methylTetrazine molecules. The TCO and methylTetrazine-NH_2_-UiO-66 obtained were diluted and stored at −20 °C. Figure 1A illustrates the synthesis of CLICK-MOF assemblies.Methyltetrazine-MOF and TCO-MOF were then incubated at 37 °C for 1 h at different volume ratios to form the MOF assembly, and its effect on fluorescence signal amplification was studied. The results are shown in Figure 2.

### 2.3. Preparation of TCO-Ab_2_

The TCO-PEG4-NHS Ester was dissolved to a concentration of 10 mM in DMF, and goat anti-mouse secondary IgG was diluted with antibody diluent. The ligand (TCO-PEG4-NHS Ester) and the ammonia-containing protein (goat anti-mouse secondary IgG, Ab_2_) were then mixed in a 20:1 molar ratio and subjected to a shock response at 37 °C for 1 h. After the reaction, 50 mM Tris-HCl (pH = 8.0) was added to stop the reaction for 10 min. The mixture was centrifuged at 10,000 rpm for 20 min at 4 °C using a clean ultrafiltration tube (30 kDa) and washed three times with PBS (pH 7.4, 10 mM) to remove excess TCO molecules. Finally, the solution was diluted to 2 mg/mL and stored at −20 °C.

### 2.4. Detection Process of CLICK-FLISA

The ZEN and FB1 complete antigens were coated onto a 96-well black enzyme-linked plate using a CBS buffer at a ratio of 1:4000, with 100 μL per well, and incubated at 4 °C for 12 h. A 1% BSA sealing solution was then added at 150 μL per well, and the plate was incubated at 37 °C for 1 h. Subsequently, ZEN and FB1 standard solutions, diluted to different concentrations, were added at 50 μL per well. Then, ZEN and FB1 monoclonal antibodies, diluted with antibody diluent at ratios of 1:8000 to 1:4000, were added at 50 μL per well and incubated at 37 °C for 1 h. A negative control group without the standard substance was included. A pre-prepared TCO secondary antibody, diluted to 100 μL per well, was then added and incubated at 37 °C for 1 h. The prepared Click-MOFs assembly (2 mg·mL^−1^) was added at 100 μL per well and incubated at 37 °C for another hour. PBST buffer was used for washing between each step. Fluorescence values were measured using a SpectraMax M5 multifunctional microplate reader, purchased from Molecular Devices Co., Ltd. (Shanghai, China), with an excitation wavelength of 324 nm and an emission wavelength of 450 nm, utilizing the “top reading” mode.

### 2.5. Selectivity for ZEN and FB1

**Figure 3:**
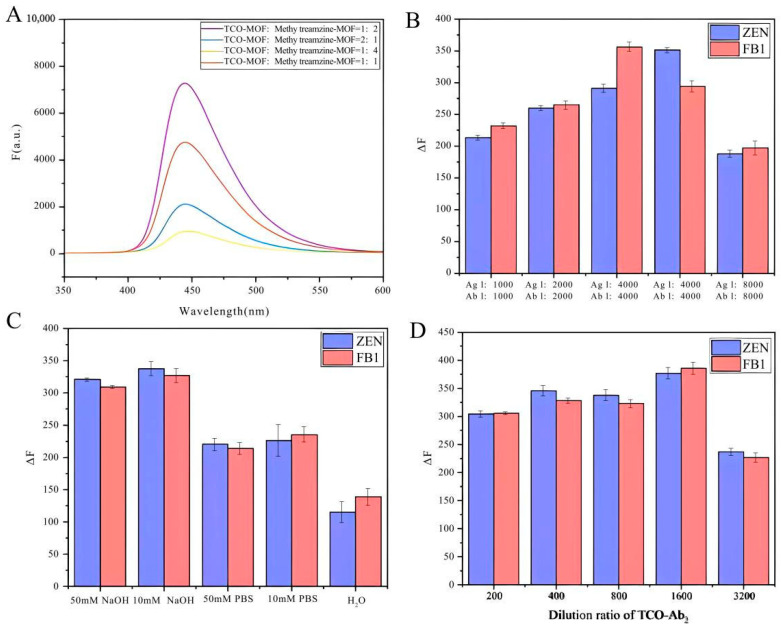
Optimization of detection performance of CLICK-FLISA. (**A**) Molar ratio of TCO-MOF and methylTetrazine-MOF, (**B**) dilution ratio of antigen and antibody, (**C**) the category of buffer, and (**D**) dilution ratio of TCO-Ab_2_.

The specificity of CLICK-FLISA for the detection of two mycotoxins was investigated. AFM1, AFB1, DON, and OTA were selected as representative mycotoxins commonly produced after maize mildew. As shown in Figure 3, high concentrations (50 ng·mL^−1^) of AFM1, AFB1, DON and OTA standards were added, and the specificity of CLICK-FLISA was verified by comparing fluorescence intensity.

### 2.6. Actual Sample Detection

Sample pretreatment: 25 g of supermarket corn flour was weighed into a beaker, and 50 mL of a 70% methanol solution was added. The mixture was stirred using a magnetic stirrer for 5 min. After filtration, 10 mL of clear liquid was taken, diluted 10 times with pure water, and filtered with a 0.45 μm filter membrane. ZEN and FB1 standard solutions with different concentrations were added to the filtrate for actual sample recovery.

Determination of spiked recovery: Add ZEN and FB1 standards in different concentrations to the sample and calculate the spiked recovery of the sample. The calculation method is as follows:

Spiked recovery (%) = sample tested value/added value × 100%.

**Figure 4:**
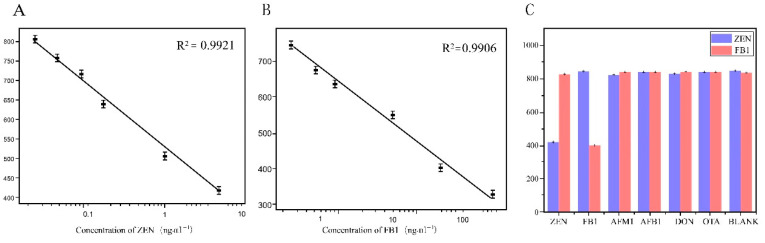
Sensitivity and selectivity of CLICK-FLISA strategy. (**A**) Linear standard curve of ZEN detection by CLICK-FLISA. (**B**) Linear standard curve of FB1 detection by CLICK-FLISA. (**C**) Selectivity of this strategy for the detection of ZEN and FB1.

Each concentration is measured 3 times. The results are shown in Figure 4.

## 3. Results and Discussion

### 3.1. Mechanisms Associated with CLICK-FLISA

The assembly mechanism of CLICK-FLISA and the detection principle are illustrated in Figure 1. To address the issues of high background values due to poor fluorescence stability and a non-significant Stokes shift, the MOF material NH_2_-UiO-66 with an excitation wavelength of 324 nm and an emission wavelength of 450 nm was utilized. The selection of 2-aminoterephthalic acid as the ligand is crucial. Its amino functionality endows the MOF with the potential for further chemical modifications, particularly in attaching fluorescent probes. Additionally, the presence of the amino group influences the electronic properties of the MOF, thereby enhancing photoluminescence (PL) intensity.

This material was assembled using click chemistry with tetrazine-trans cyclooctene, amplifying the fluorescence signal and improving FLISA sensitivity (Figure 1A). This method is not only efficient and catalyst-free but also ensures that the fluorescent probes are securely anchored on the MOF surface. The resulting MOF assemblies significantly enhance the stability and intensity of the photoluminescence (PL) signal. The high specificity of click chemistry reduces nonspecific binding and background fluorescence, thereby increasing the signal-to-noise ratio and enhancing the sensitivity of the FLISA. Fluorescent labeling was achieved by coupling the MOF with the secondary antibody using the same modification method (Figure 1B). The click chemistry ligand activated by N-hydroxysuccinimide (NHS) facilitated coupling with NH_2_-UiO-66 and the antibody, improving reaction kinetics without needing a catalyst and ensuring excellent biocompatibility. Finally, four experimental conditions were optimized, resulting in the CLICK-FLISA method capable of detecting multiple targets with a multifunctional microplate reader. This method successfully demonstrated the simultaneous and sensitive detection of ZEN and FB1, as shown in Figure 1C.

### 3.2. Feasibility Verification and Characterization of the CLICK-FLISA System

The MOFs were characterized in detail. Figure 2A showed the X-ray diffraction (XRD) spectra of UiO-66 and NH_2_-UiO-66. Typical Bragg peaks are located at 2θ = 7.37°, 8.54°, 14.85°, 17.40° and 25.51°. These peak values are in good agreement with previous reports [35,36], indicating that UiO-66 and NH_2_-UiO-66 were successfully synthesized and share the same crystal structure. Furthermore, the functional groups present in the structure of NH_2_-UiO-66 were determined by FT-IR analysis, as shown in Figure 2B. The characteristic peak range of the FT-IR spectrum was set as 500–4000 cm^−1^. The absorption bands at lower frequencies (573, 654 and 787 cm^−1^) were due to Zr-(OC) symmetric stretching, O-H bending, and Zr-O bond vibration, respectively [37]. Additionally, strong peaks at 1367 and 1424 cm^−1^ were observed, corresponding to the stretching patterns of the C-N bond and carboxylic acid group, respectively [38,39]. The peaks at 1573 and 1690 cm^−1^ indicate the presence of C=C and C=O (carbonyl) functional groups in the synthesized MOF [40]. Finally, weak peaks at 3340 and 3420 cm^−1^ were considered proof of the presence of the amino group [41]. The morphology of NH_2_-UiO-66 was examined using scanning electron microscopy (SEM) and transmission electron microscopy (TEM). As shown in Figure 2C, SEM showed that NH_2_-UiO-66 presents regular octahedral crystals. In order to further illustrate the morphology of NH_2_-UiO-66, TEM was shown in Figure 2D, and the morphology of the image was similar to SEM. The average particle size of NH_2_-UiO-66 was about 400 nm. TEM was also used to characterize the CLICK-MOF assembly. As seen in Figure 2E, NH_2_-UiO-66 was successfully assembled, maintaining its regular octahedral structure. The conjugation of antibodies and click ligands is a key step in this experiment. The goat anti-mouse secondary IgG and the antibody conjugated with the click ligand were identified using protein gel electrophoresis. The results, shown in Figure 2F, where M is the Marker, band 1 is the protoantibody, and band 2 is the antibody conjugated with the click ligand, indicate successful conjugation. Compared with band 1, band 2 shows a significant upward shift with less overlap, confirming that the antibodies were successfully conjugated with the click ligand and can be used in subsequent experiments.

### 3.3. Optimization of the Analytical Performance of the CLICK-FLISA System

The assembly volume ratio of CLICK-MOFs (CLICK) was optimized, and the correlation between changes in fluorescence signal intensity and variations in the molar ratio of TCO-MOF to methylTetrazine-MOF was studied first.

As shown in Figure 3A, the fluorescence signal intensity reaches its peak at a TCO-MOF to methylTetrazine-MOF ratio of 1:2. This optimal ratio likely provides the most favorable stoichiometric balance, enhancing the efficiency of the click chemistry reaction. Consequently, the reaction between TCO and methylTetrazine proceeds more completely, forming a more stable MOF–antibody complex and thus amplifying the fluorescence signal. Additionally, the higher concentration of methylTetrazine-MOF ensures more successful binding of TCO-modified antibodies to the MOF. This implies that a greater number of antibodies can be effectively immobilized on the MOF, boosting the specific fluorescence signal. Conversely, at a TCO-MOF to methylTetrazine-MOF ratio of 1:4, the signal diminishes, likely due to steric hindrance effects and increased non-specific binding. Next, the concentration of antigen and antibody and the concentration of goat anti-mouse second IgG modified with click ligand were optimized. As shown in Figure 3B,D, it was observed that lower dilution ratios resulted in higher experimental background values, whereas higher dilution ratios led to reduced MOF binding and fluorescence values. Specifically, the optimal dilution ratios for antigen, antibody, and TCO-labeled secondary antibody were 4000, 8000, and 1600, respectively. Finally, under optimized conditions, five different buffers—50 μM NaOH, 10 μM NaOH, 50 mM PBS, 10 μM PBS, and pure water—were added to the neimengu film for final detection. As shown in Figure 3C, the fluorescence signal obtained with NaOH as the buffer is higher than that obtained with the other three buffer conditions. The enhanced fluorescence signal was due to the hydrolysis of NH_2_-UiO-66 in an alkaline medium, releasing a large amount of the fluorescent ligand NH_2_·H_2_BDC.

### 3.4. Evaluation of the Analytical Performance of the CLICK-FLISA System

Under optimal experimental conditions, the CLICK-FLISA method based on a metal–organic skeleton was established for the detection of two mycotoxins in food. In this experimental method, higher toxin concentrations lead to more binding to the antigen coated on the well plate, resulting in less binding of the monoclonal antibody to the antigen [42]. After elution, the binding amount of TCO-Ab_2_, which specifically identifies monoclonal antibodies, also decreases correspondingly, leading to less binding of the MOF on the 96-well plate. Consequently, lower fluorescence intensity corresponds to higher toxin concentrations. According to the fluorescence intensity corresponding to different concentrations of toxin, the standard curve was established with Log10 of different diluted concentrations of toxin standard as the horizontal coordinate and the corresponding fluorescence intensity value as the vertical coordinate. The linear range of ZEN detected by this detection method was 0.02~5 ng·mL^−1^. The linear equation was and the detection limit was 0.0161 ng·mL^−1^ by calculation. The linear range of FB1 detection was 0.4~250 ng·mL^−1^; the linear equation was

y = −148.73584Logx + 679.85186, R^2^ = 0.9906,

and the detection limit was 0.394 ng·mL^−1^. The relevant data are shown in Figure 4A,B.

As shown in Figure 4C, The specificity of CLICK-FLISA for the detection of two mycotoxins was investigated. Fluorescence intensity values for ZEN (5 ng·mL^−1^) and FB1 (10 ng·mL^−1^) were notably lower compared to AFM1, AFB1, DON, and OTA, indicating significant fluorescence inhibition only in the presence of ZEN and FB1. Conversely, other interferents like AFM1, AFB1, DON, and OTA showed minimal fluorescence inhibition, even at higher concentrations. The signal of the ImageJ responds in the presence(a) or absence(b) of the target are shown in Figure S2. The aforementioned results clearly demonstrate that the CLICK-FLISA method developed in this study exhibits excellent specificity, attributed to the specific antigen–antibody binding. This lays a solid foundation for subsequent experiments in complex real sample matrices.

To validate the practicality of the CLICK-FLISA method based on metal–organic frameworks in real-world detection, standard toxin solutions were added to cornmeal samples, with each measurement repeated five times. The results are shown in Table 1. At the concentration of 0.05~2 ng·mL^−1^, ZEN exhibited recovery rates ranging from 88% to 117.1% at concentrations of 0.05 ng·mL^−1^ to 2 ng·mL^−1^, with relative standard deviations (RSDs) between 4.8% and 9.2%. At the same time, FB1 recoveries ranged from 96.8% to 115.6% at concentrations from 1 ng·mL^−1^ to 100 ng·mL^−1^, with RSDs ranging from 2.1% to 6.7%. These results indicate high detection accuracy, suggesting potential application in rapid maize detection. Additionally, this method was compared with the FITC-based competitive fluorescence immunoassay (FLISA) shown in Figure 2, demonstrating higher sensitivity in labeled sample groups compared to the FLISA using fluorescein as a tracer. The schematic diagram of the FITC-based FLISA is provided in Figure S3. Furthermore, the linear standard curves for the detection of ZEN and FB1 using CLICK-FLISA are also shown in Figure S3. As shown in Table 2, comparing parameters such as detection range and detection limits for the same toxins with those of ELISAs, LFIAs, and other methods indicates that the method proposed in this study exhibits superior detection capabilities.

## 4. Conclusions

In traditional immunoassays, antigens or antibodies are usually labeled with one or more signaling molecules, which greatly limits sensitivity [43]. The introduction of nano-carriers is an effective way to improve the sensitivity of an immunoassay. This approach allows for a large number of signal molecules to be uploaded, resulting in a higher signal than traditional strategies [44]. In this experiment, NH_2_-UiO-66 was selected as the fluorescent probe, and click chemistry was selected as the coupling segment. Based on the innovation of the detection method, the detection signal was significantly amplified. It could be proven that this study developed a detection method with broad application prospects for the actual sample analysis of rapid on-site detection.

## Figures and Tables

**Table 1:** Actual sample testing and labeling recovery (*n* = 5).

Sample	Target	Fortified Concentration	Detection Value	Recovery (%)	RSD (%)
1	ZEN	0.05	0.043	86.0	8.4
2	ZEN	0.1	0.105	105.1	8.2
3	ZEN	0.5	0.578	115.6	5.8
4	ZEN	1	1.168	116.8	9.2
5	ZEN	2	2.226	111.3	4.9
6	FB1	1	1.150	115.0	6.1
7	FB1	5	4.855	97.1	6.7
8	FB1	10	10.914	109.1	5.8
9	FB1	50	53.352	106.7	3.4
10	FB1	100	104.340	104.3	2.3

**Table 2:** Comparison of three detection methods.

Method	Target	Detection Range (ng·mL^−1^)	LOD (ng·mL^−1^)	Recovery (%)	RSD (%)
ELISA	ZEN	3.9~2000	3.228	-	-
	FB1	40~4000	22.787	-	-
FLISA	ZEN	0.2~250	0.124	92~107.9	2.7~5.6
	FB1	12~2500	2.103	91.2~107.9	2.7~7.3
LFIA	ZEN	0.8~40	0.70	88.28~104.68	-
	FB1	4~80	3.27	88.36~112.49	-
CLICK-FLISA	ZEN	0.02~5	0.016	88~117.1	4.8~9.2
	FB1	0.4~250	0.394	96.8~115.6	2.1~6.7

## Data Availability

Data are contained within the article.

## Supplementary Materials

The following supporting information can be downloaded at https://www.mdpi.com/article/10.3390/bios14070355/s1, Chemical equation and IEDDA reaction principle. Specific binding of antigen and antibody. Schematic of the FLISA with FITC as tracer. Comparison between a competitive ELISA, fluorescence immunoassay based on FITC, and CLICK-FLISA based on metal–organic frameworks.
