# Supplementary material for: CLICK-FLISA Based on Metal–Organic Frameworks for Simultaneous Detection of Fumonisin B1 (FB1) and Zearalenone (ZEN) in Maize
Source: Biosensors (Basel). 2024 Jul 21;14(7):355. doi: 10.3390/bios14070355 (PMC11275017; doi:10.3390/bios14070355)
Supplement: Supplementary file 1 [file biosensors-14-00355-s001.zip › biosensors-3064740-supplementary.pdf]

biosensors

Supporting Information

CLICK-FLISA Based on Metal–Organic Frameworks for Simultaneous Detection of Fumonisin B1（FB1) and Zearalenone (ZEN) in Maize

Jingyang Zhang †, Banglei Zhu †, Xiaoyu Zhang †, Yuan Peng, Shuang Li, Dianpeng Han, Shuyue Ren, Kang Qin, Yu Wang *, Huanying Zhou * and Zhixian Gao *

Military Medical Sciences Academy, Academy of Military Sciences, Tianjin 300050, China; 15760527290@163.com (J.Z.); z1095317236@163.com (X.Z.); dalidao@139.com (Y.P.); liza3320@163.com (S.L.); 15210520025@126.com (D.H.); renshuyue2018@163.com (S.R.); qinkang2020@foxmail.com (K.Q.)

* Correspondence: wangyuyu9210@163.com (Y.W.); zhouhytj@163.com (H.Z.); gaozhx@163.com (Z.G.)

† These authors contributed equally to this work.

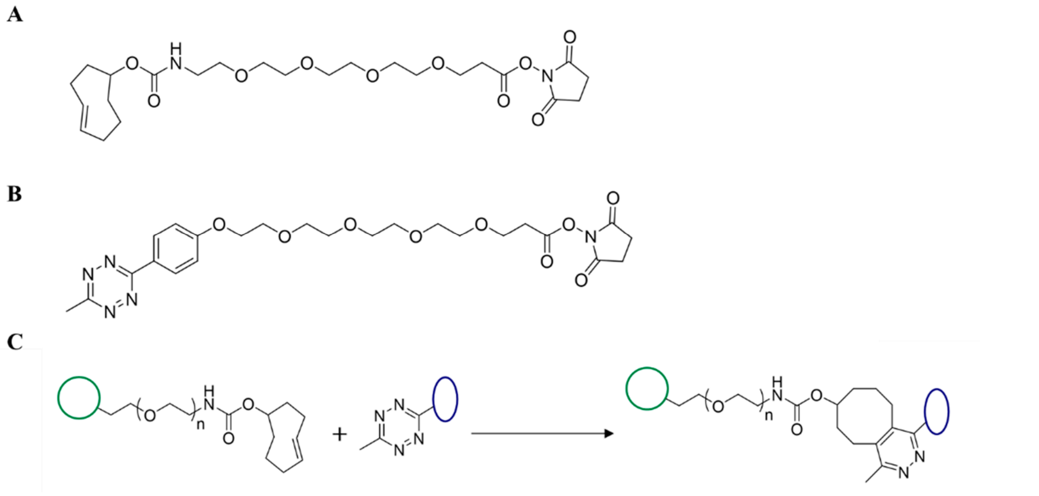

Figure S1. Chemical equation (A) TCO-PEG4-NHS Ester (B) methyltetrazine-PEG4-NHS Ester (C) reaction principle.

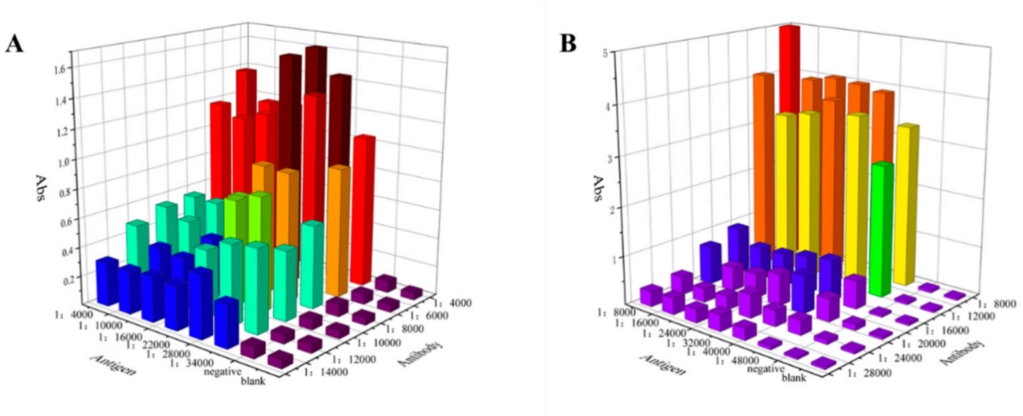

Figure S2. The signal of the ImageJ responds in the presence(a) or absence(b) of the target.

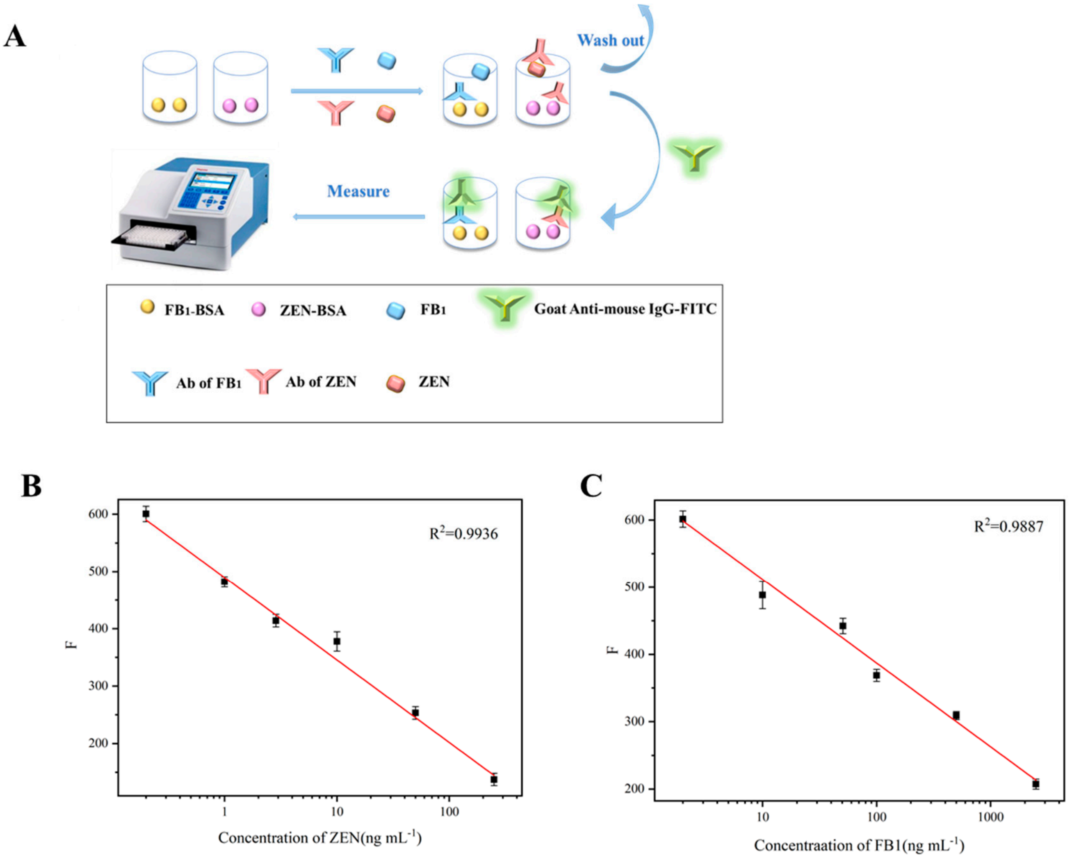

Figure S3. (A)Schematic of FLISA with FITC as tracer. Linear standard curve of (B)ZEN and (C)FB1 detection by CLICK-FLISA.

Disclaimer/Publisher's Note: The statements, opinions and data contained in all publications are solely those of the individual author(s) and contributor(s) and not of MDPI and/or the editor(s). MDPI and/or the editor(s) disclaim responsibility for any injury to people or property resulting from any ideas, methods, instructions or products referred to in the content.
